# ^131^I SPECT/CT provides prognostic information in patients with differentiated thyroid cancer

**DOI:** 10.1007/s00259-025-07187-1

**Published:** 2025-03-15

**Authors:** Marieke Heinrich, Elias Blickle, Philipp E. Hartrampf, Natalie Hasenauer, Aleksander Kosmala, Alexander Kerscher, Nicolas Schlegel, Frederik A. Verburg, Andreas K. Buck, Kerstin Michalski

**Affiliations:** 1 Department of Nuclear Medicine, University Hospital Wuerzburg, Wuerzburg, Germany; 2 Comprehensive Cancer Centre Mainfranken, University Hospital Wuerzburg, Wuerzburg, Germany; 3 Department of Surgery I, University Hospital Wuerzburg, Wuerzburg, Germany; 4 Department of Radiology and Nuclear Medicine, Erasmus Medical Centre, University Rotterdam, Rotterdam, Netherlands

**Keywords:** Lymph node metastases, Complete response, Progression-free survival, Reoperation, Radioiodine therapy, ^131^I SPECT/CT

## Abstract

**Purpose:**

The aim of this study is to investigate the impact of lymph node metastases (LNM) detected on cervical ^131^I single photon emission computed tomography/computed tomography (SPECT/CT) after the first radioiodine therapy (RAI) on complete response (CR) and progression-free survival (PFS) in patients with differentiated thyroid cancer (DTC).

**Methods:**

This retrospective study included 942 DTC patients who underwent cervical ^131^I SPECT/CT after their first RAI. LNM were categorized based on CT (enlarged ≥ 1 cm, small < 1 cm) and ^131^I uptake. CR and PFS were analysed using Kaplan–Meier curves and Cox regression.

**Results:**

Patients with no LNM had a shorter median time to CR (9.4 months) than those with LNM (44 months, HR 2.2; *p* < 0.01) and a lower risk of progression (median PFS not reached, HR 0.46; *p* < 0.01). Among patients with LNM, those with enlarged ^131^I negative LNM had the longest time to CR (24 months, HR 0.36; *p* < 0.01). Patients with small LNM had a PFS similar to patients without LNM (median PFS not reached, HR 1.22; *p* = 0.54). Reoperation after first RAI (13.5 months) led to earlier CR than second RAI (median not reached) in patients with enlarged LNM. For small LNM, second RAI was associated with longer PFS than reoperation (38.4 months vs. not reached, HR 4.0; *p* = 0.02).

**Conclusion:**

Patients without LNM on post-therapy ^131^I SPECT/CT have better chances for early CR and longer PFS. Patients with LNM benefit from early reoperations but treatment strategies should be tailored based on LNM characteristics.

**Supplementary Information:**

The online version contains supplementary material available at 10.1007/s00259-025-07187-1.

## Introduction

In differentiated thyroid cancer (DTC), the presence of cervical lymph node metastases (LNM) is an adverse prognostic factor which has a marked adverse impact on both the chance of achieving complete remission (CR) of disease and recurrence-free survival as well as DTC specific mortality rates, especially in patients ≥ 55 years [1–5].

LNM can be found preoperatively by clinical examination or ultrasound, during surgery, or postoperatively during histopathological work-up of the surgical specimen as well as on post-therapy scanning after radioiodine therapy with ^131^I (RAI). For RAI post-therapy scanning, the integration of single photon emission computed tomography (SPECT) in combination with computed tomography (CT) greatly enhances sensitivity and specificity and should therefore be performed in addition to planar whole body scans [6].

Especially for LNM detected on RAI post-therapy scans, there is ongoing debate about their impact on prognosis and the effectiveness of further treatment [7]. Several studies analysed the value of a second RAI after reoperation and showed inconsistent findings [8–10]. However, to the best of our knowledge, no studies compared the impact of LNM size in combination with ^131^I uptake and further treatment on prognosis in terms of achieving CR and progression rates, nor does it contain studies comparing whether reoperation or RAI alone would have a greater positive impact on prognosis.

Hence, the aim of the present study is to investigate the effect of postoperative LNM detected on ^131^I SPECT/CT, considering ^131^I uptake and size, on achieving CR and progression-free survival (PFS). This analysis will be conducted for the entire patient cohort as well as subgroups based on subsequent treatment modalities, namely reoperation or RAI.

## Methods

### Patient cohort

We retrospectively analysed all patients with DTC and LNM who were treated with RAI and underwent post therapeutic ^131^I SPECT/CT at our department. We excluded patients with distant metastases. The local Ethics Committee waived the need for further approval due to the retrospective character of the study (waiver no. 20231103 02). In accordance with ATA guidelines from 2015, CR was defined as TSH-stimulated thyroglobulin (Tg) ≤ 1 ng/ml and the absence of ^131^I uptake on diagnostic whole-body scan during TSH-stimulation. We defined progression as the diagnosis of new lesions in diagnostic ^131^I scintigraphy, FDG-PET/CT or ultrasound with confirmation using fine needle aspiration or the occurrence of a doubling of Tg levels at similar TSH levels before achieving CR or any increase in Tg levels after achieving CR.

### Image evaluation

**Fig. 1:**
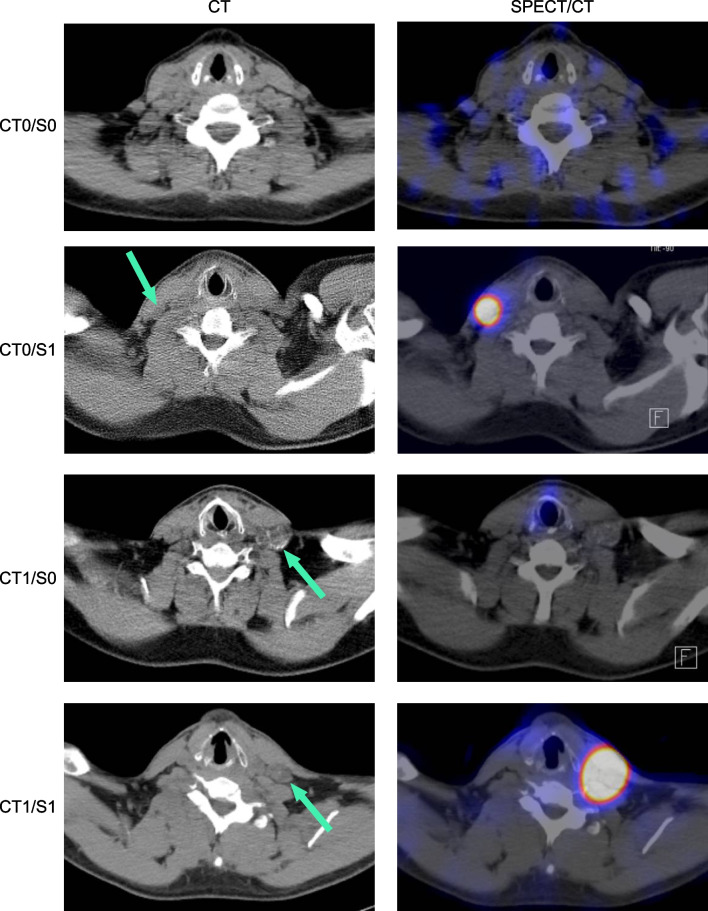
CT (left column) and fusion of SPECT and CT (right column) of a first RAI in the subgroups CT0/S0, CT0/S1, CT1/S0 and CT1/S1. LNM are marked with a green arrow in CT. Abbreviations: CT: computer tomography; RAI: radioiodine therapy; SPECT: single photon emission computer tomography

^131^I SPECT/CT images were analysed visually as part of the clinical routine by one experienced nuclear medicine specialist. A lymph node with a short axis diameter ≥ 1 cm was defined as”enlarged”, under 1 cm as small. Pathological ^131^I uptake was defined as visually discernible uptake exceeding the uptake in the salivary glands. To evaluate the nodal status on ^131^I SPECT/CT we defined four groups: CT0/S0 for anatomical normal lymph nodes and no visual ^131^I uptake, CT0/S1 for anatomical normal lymph nodes and increased ^131^I uptake. CT1/S0 for anatomical pathological enlarged lymph nodes without ^131^I uptake and CT1/S1 for enlarged lymph nodes with pathological ^131^I uptake, see Fig. 1*.*

### Further treatment

All patients were treated with thyroidectomy and initial RAI followed by thyroid-stimulating hormone (TSH) suppression with Levothyroxine. The activity amount for all RAI treatments was determined based on risk adaptation, in accordance with the recommendations of SNMMI and EANM for differentiated thyroid cancer [11]. Patients with an incomplete response after first RAI received either additional RAI therapy, reoperation, or reoperation followed by additional RAI therapy as a second-line treatment. The decision for further therapy was made interdisciplinary within the framework of a tumour board for endocrine tumours.

### Follow up

Six months after each RAI, a diagnostic whole-body scan with 300 MBq ^131^I under TSH stimulation was performed. This was followed by semi-annual check-ups using ultrasound and Tg measurement under TSH suppression. After five years, the check-ups were conducted annually. If any abnormalities were detected during follow-up, additional imaging modalities such as whole-body scintigraphy or PET/CT or fine needle aspiration were used for further diagnostics. The first evaluation of a complete response occurred during the diagnostic whole-body scan under TSH-stimulation six months after the first RAI. Progression or recurrence was evaluated at each follow-up examination.

### Statistical analysis

We used GraphPad Prism version 9.3.0 (GraphPad Software, San Diego, California, United States) for statistical analyses. Unless otherwise described data are presented in median and range or with their 95% confidence interval (CI) in parentheses. Time to complete response and time to progression or recurrence was calculated starting with the day of diagnosis. We used Kaplan–Meier-Curves, log-rank comparisons and the Cox proportional hazard to analyse the chance for CR, the risk of progression or recurrence and the influence of different therapies after first RAI on CR and PFS.

## Results

### Patient characteristics

Out of 3298 patients with DTC, we identified 1009 patients with LNM and ^131^I SPECT/CT after initial RAI. We excluded 67 patients with distant metastasis, either discovered before or on ^131^I SPECT/CT, resulting in a cohort of 942 patients. These patients were initially diagnosed with DTC between November 2006 and September 2023. The median follow up period was 51 months (1 – 209 months). All 942 patients underwent one- or two-staged total thyroidectomy and initial RAI including post therapeutic ^131^I SPECT/CT. For all patients’ characteristics, see Table 1 and for further information regarding surgery prior to initial RAI see supplemental Table 1.

**Table 1:** Patients characteristics *n* = 942

		Median [years]	Range
Age at diagnosis	all	48	18–89
		number [n]	percentage [%]
Age at diagnosis	< 55 years	651	69
	≥ 55 years	291	31
Female		660	70
Male		282	30
		number [n]	percentage [%]
Histology	papillar	797	85
	follicular	145	15
		number [n]	percentage [%]
T classification according to the revised reprint 2020 of the 8th edition of the TNM classification of malignant tumours	1	475	50
	2	212	23
	3	235	25
	4	13	1
	x	7	1
		number [n]	percentage [%]
Surgeries previous to first RAI	1	618	65.5
	2	321	34
	3	3	0.5
		median [GBq]	range
initial RAI	^131^I activity	3.6	1.01 – 12.57
		median [days]	95% CI
time diagnosis—RAI		33	32 −34
time surgery—RAI		32	31—33
		number [n]	percentage [%]
Complete response	yes	744	79
	no	198	21
Progression	yes	79	8
	no	863	92

*RAI* radioiodine therapy

### Complete response and progression based on ^131^I SPECT/CT

**Fig. 2:**
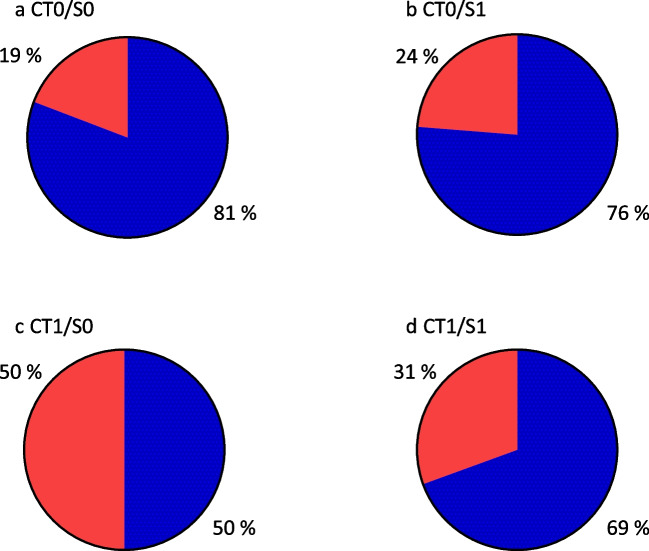
Patients with complete response (blue) versus patients without complete response (red) in the subgroups of patients with no evidence of LNM (**a** CT0/S0), patients with small ^131^I positive LNM (**b** CT0/S1), patients with enlarged ^131^I negative LNM (**c** CT1/S0) and patients with enlarged ^131^I positive LNM (**d** CT1/S1). Abbreviations: CT: computer tomography; LNM: lymph node metastases; RAI: radioiodine therapy; S: single photon emission computer tomography

745 of 942 patients were classified CT0/S0, 139 patients CT0/S1, 22 patients CT1/S0 and 36 patients CT1/S1. All subgroups showed more female than male patients. All subgroups reached the median time to CR, see Table 2 for further information. Complete response rates were 81%, 76%, 50% and 69% for CT0/S0, CT0/S1, CT1/S0 and CT1/S1 (Fig. 2). The median PFS was not reached in CT0/S0, CT0/S1 and CT1/S0. Patients classified CT1/S1 suffered progression with a median PFS of 93.4 months (Table 2).

**Table 2:** Complete response and progression depending on ^131^I SPECT/CT-based classification. Presence of complete response and progression in the subgroups of patients with no evidence of LNM in ^131^I SPECT/CT (CT0/S0), patients with small ^131^I positive LNM (CT0/S1), patients with enlarged ^131^I negative LNM (CT1/S0) and patients with enlarged ^131^I positive LNM (CT1/S1)

	CT0/S0	CT0/S1	CT1/S0	CT1/S1
Patients [n]	745	139	22	36
Complete response				
Yes [n] (%)	602 (81)	106 (76)	11 (50)	25 (70)
No [n] (%)	143 (19)	33 (24)	11 (50)	11 (30)
Median time to CR [months]	9.4	11.6	24.0	12.9
Progression				
Progression and Recurrence [n] (%)	50 (7)	12 (9)	6 (27)	11 (31)
Progression [n] (%)	38 (5)	8 (6)	5 (23)	10 (28)
Recurrence [n] (%)	12 (2)	4 (3)	1 (5)	1 (3)
Median PFS [months] (range)	Not reached	Not reached	Not reached	93.4

*CR* complete response, *CT* computer tomography, *LNM* lymph node metastases, *PFS* progression free survival, *RAI* radioiodine therapy, *S/SPECT* single photon emission computer tomography

**Fig. 3:**
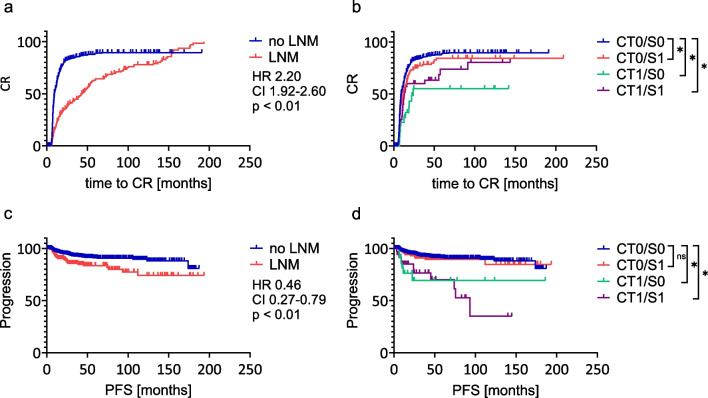
Time to CR (**a**) and PFS (**c**) in patients with no evidence of LNM in ^131^I SPECT/CT (CT0/S0 blue) and patients with evidence of LNM in ^131^I SPECT/CT (CT0/S1, CT1/S0, CT1/S1; red). Time to CR (**b**) in patients with no evidence of LNM in ^131^I SPECT/CT (CT0/S0 blue), compared to patients with small ^131^I positive LNM (CT0/S1 red; HR 0.81; 95% CI 0.67 – 0.98; *p* = 0.04), patients with enlarged ^131^I negative LNM (CT1/S0 green; HR 0.36; 95% CI 0.25 – 0.51; *p* < 0.01), and patients with enlarged ^131^I positive LNM (CT1/S1 purple; HR 0.58; 95% CI 0.42 – 0.80; *p* < 0.01). PFS (**d**) in the subgroups CT0/S0 (blue), CT0/S1 (red; HR 1.22; 95% CI 0.62–2.38; *p* = 0.54), CT1/S0 (green; HR 4.38 95% CI 0.87–22.23; *p* < 0.01) and CT1/S1 (purple; HR 5.19; 95% CI 1.46–18.49; *p* < 0.01). Abbreviations: CR: complete response CT: computer tomography; HR: Hazard ratio; LNM: lymph node metastases; PFS: progression free survival; S/SPECT: single photon emission computer tomography

Patients without evidence of LNM in the ^131^I SPECT/CT of the initial RAI (CT0/S0, *n* = 745) showed a significant higher chance (HR 2.20; 95% CI 1.92 – 2.60; *p* < 0.01) to reach CR than patients with LNM (CT0/S1, CT1/S0 and CT1/S1, *n* = 179) and a shorter time to CR (9.4 months vs 44 months; Fig. 3a). Referring to progression, Patients classified CT0/S0 showed a significant lower risk for progression (HR 0.46; 95% CI 0.27 – 0.79; *p* < 0.01) than patients with LNM in ^131^I SPECT/CT (CT0/S1, CT1/S0 and CT1/S1, *n* = 179; Fig. 3c). Therefore, we chose CT0/S0 as a reference group.

Analysis of the time to CR across subgroups revealed that CT1/S0 had the longest median time to CR with 24 months (HR 0.36; 95% CI 0.25–0.51; *p* < 0.01), and the lowest probability of achieving CR compared to the reference group (CT0/S0), with 9.4 months (Fig. 3b). The subgroup CT1/S1 had a median time to CR of 12.9 months (HR 0.58; 95% CI 0.42–0.80; *p* < 0.01). The chance of reaching CR for CT0/S1 was closest to that of the reference group, with a median time to CR of 11.6 months (HR 0.81; 95% CI 0.67–0.98; *p* = 0.04). CT1/S0 demonstrated a significantly lower chance of achieving CR (HR 0.44; 95% CI 0.28–0.70; p < 0.01) compared to CT0/S1. No significant difference was observed between CT1/S1 and CT0/S1 (HR 0.72; 95% CI 0.48–1.07; p = 0.13) or between CT1/S0 and CT1/S1 (HR 0.87; 95% CI 0.31–1.16; *p* = 0.15). Figure 3b illustrates the time to CR across all subgroups.

For PFS, CT1/S0 (median PFS not reached) showed a shorter PFS than the reference group (HR 4.38; 95% CI 0.87–22.23; *p* < 0.01; Fig. 3d). The subgroup CT1/S1 showed a shorter PFS too (median PFS 93.4 months; HR 5.19; 95% CI 1.46–18.49; *p* < 0.01). CT0/S1 showed a similar risk to the reference group (median PFS not reached, HR 1.22; 95% CI 0.62–2.38; *p* = 0.54). Both CT1/S0 (HR 3.67; 95% CI 0.89–15.22; *p* < 0.01) and CT1/S1 (HR 4.18; 95% CI 1.44–12.13; *p* < 0.01) demonstrated a significantly higher risk for progression compared to CT0/S1. However, no significant difference in the risk for progression was found between CT1/S0 and CT1/S1 (HR 1.13; 95% CI 0.42–3.01; *p* = 0.81). Figure 3d shows PFS across all subgroups.

### Treatment decision based on ^131^I SPECT/CT

**Fig. 4:**
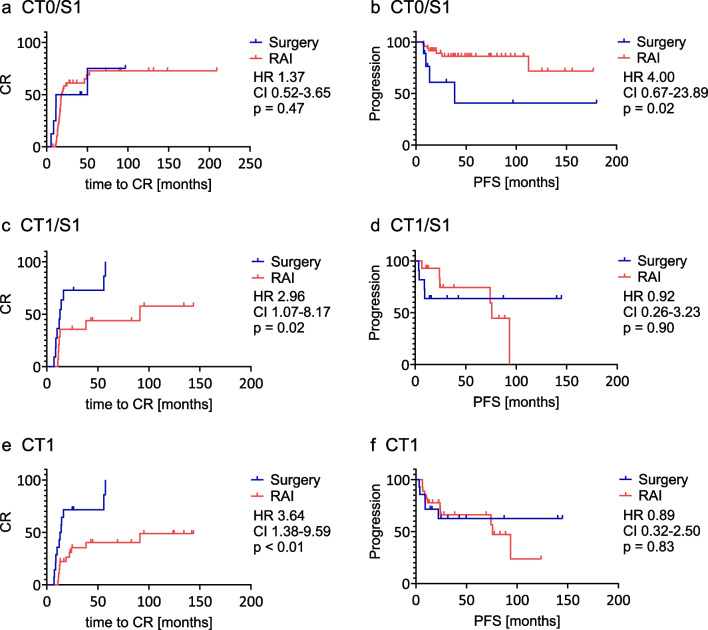
Treatment after first RAI in patients with small ^131^I positive LNM (CT0/S1; **a**-**b**), patients with enlarged ^131^I positive LNM (CT1/S1; **c**-**d**) and in patients with enlarged LNM (CT1; **e**–**f**). Time to CR (**a**; **c**; **e**) and PFS (**b**; **d**; **f**) in the groups Reoperation (blue) and second RAI (red). Abbreviations: CR: complete response; CT: computer tomography; HR: Hazard ratio; LNM: lymph node metastases; PFS: progression free survival; RAI: radioactive iodine; S: single photon emission computer tomography

27 patients received reoperation, 19 with adjuvant RAI. 302 patients received second RAI and 613 needed no further therapy. The frequency of reoperation, RAI and no further therapy in the subgroups can be found in Table 3*.* The detection of LNM in ^131^I SPECT/CT was the indication for all reoperations in the subgroups with LNM (CT0/S1; CT1/S0; CT1/S1). The detection of LNM on ^131^I SPECT/CT was followed by a second RAI in 51% of patients in the subgroup CT0/S1 and in 64% of patients in the subgroup CT1/S1. In the other cases, the indication for further RAI was thyroid remnant on diagnostic whole body scintigraphy or not sufficiently dropped stimulated Tg level. For further information see supplemental Table 1. In the subgroup CT0/S1, the median time to CR did not differ significantly between patients with reoperation (30.9 months) and patients with second RAI (18.1 months; HR 1.37; CI 0.52 – 3.65; *p* = 0.47; Fig. 4a). The time to progression was significantly shorter in patients with reoperation (38.4 months) than in patients with second RAI (not reached; HR 4.00; 95% CI 0.67 – 23.89; *p* = 0.02; Fig. 4b).

**Table 3:** Treatment decision after first RAI. Treatment decision after first RAI in the subgroups of patients with no evidence of LNM on ^131^I SPECT/CT (CT0/S0), patients with small ^131^I positive LNM (CT0/S1), patients with enlarged ^131^I negative LNM (CT1/S0) and patients with enlarged ^131^I positive LNM (CT1/S1)

		CT0/S0	CT0/S1	CT1/S0	CT1/S1
Patients [n]		745	139	22	36
therapy					
Reoperation	[n] (%)	5 (1)	9 (6)	2 (9)	11 (31)
2. RAI	[n] (%)	226 (30)	49 (35)	13 (59)	14 (38)
None	[n] (%)	514 (69)	81 (59)	6 (27)	11 (31)

*CT* computer tomography, *LNM* lymph node metastases, *RAI* radioiodine therapy, *S* single photon emission computer tomography

In the group with enlarged ^131^I negative LNM (CT1/S0) only two reoperations were undertaken. Hence, no analysis of this subgroup was possible (Table 3).

In the subgroup CT1/S1, the median time to CR was significantly shorter in patients with reoperation (12.9 months) than in patients with second RAI (91.2 months; HR 2.96; 95% CI 1.07 – 8.17; *p* = 0.02; Fig. 4c). The time to progression did not differ significantly between patients with reoperation (not reached) and patients with second RAI (75.7 months; HR 0.92; 95% CI 0.26 – 3.23; *p* = 0.90; Fig. 4d). Patients with enlarged LNM, regardless of ^131^I avidity (CT1/S0 and CT1/S1; *n* = 58), had a shorter time to CR in case of a reoperation (median time to CR 13.5 months) than patients receiving a second RAI (median time to CR not reached; HR 3.64; 95% CI 1.38 – 9.59; *p* < 0.01; Fig. 4e). Regarding time to progression, no significant difference between reoperation (not reached) and second RAI (75.7 months; HR 0.89; 95% CI 0.32 – 2.50; *p* = 0.83; Fig. 4f) was found.

## Discussion

The present study represents the largest single-centre cohort study on the prognostic implication of LNM on post-therapy ^131^I SPECT/CT to date.

In this study, we analysed post-therapy ^131^I SPECT/CT following initial RAI in nearly 1000 patients with DTC. We found that patients with evidence of LNM on ^131^I SPECT/CT have a significantly higher risk for progression and lower chance for CR than those without LNM. Hugue et al. comparing 100 patients [12] and McHenry et al. investigating 227 patients with DTC [13] showed similar results in smaller cohorts.

Furthermore, our results suggest that the presence of LNM on ^131^I SPECT/CT can serve as an early risk assessment during initial RAI already before the first time point of delayed risk stratification at first diagnostic whole-body scintigraphy and first stimulated thyroglobulin level after initial RAI. Tuttle et al. analysing 588 follicular cell-derived thyroid cancer patients and Verburg et al. analysing 509 patients with DTC [14–16] implemented the delayed risk stratification. Our results suggest that further characterization of LNM, including size and ^131^I avidity, provides valuable prognostic information, because patients with small ^131^I positive LNM showed prolonged PFS, comparable to that of patients without any evidence of LNM on ^131^I-SPECT/CT. In contrast, patients with enlarged ^131^I positive and enlarged RAI ^131^I negative LNM showed shorter PFS and lower chance for CR. The association between size of LNM and progression was analysed before, but without considering ^131^I avidity. For example, Walter et al. followed 40 patients with DTC and small cervical LNM under active surveillance and found that most small LNM do not progress [17]. Jeon et al. showed in 292 DTC patients an association of increasing LNM size with earlier recurrence [18]. Additionally, our results showed a tendency for a graded reduction in the chance of CR, starting with small ^131^I positive LNM, similar to patients with no LNM, followed by a lower chance for enlarged ^131^I positive LNM. The lowest chance for CR showed enlarged ^131^I negative LNM, likely due to difficulties in locating and surgically removing these LNM. Since the LNM are not ^131^I avid, RAI is presumed to be ineffective, making reoperation the only effective treatment option. Interestingly, in our cohort, patients with enlarged ^131^I negative LNM did not exhibit late progression, unlike those with enlarged ^131^I positive LNM. This could be a statistical effect due to the smaller sample size in the enlarged ^131^I negative group. This observation is intriguing, as one would typically expect a ^131^I negative lesion to be dedifferentiated and RAI-refractory, which is usually associated with disease that is more aggressive. Due to the small sample size of this subgroup in our analysis, it stays unclear whether these LNM are potentially aggressive metastases of DTC or less dangerous, possibly reactive enlarged lymph nodes and further investigations are required to reveal their true nature.

Showing a prognostic value of a further characterization of LNM, including size and ^131^I avidity, raises the question if different further treatments lead to different outcomes in the subgroups. Therefore, we examined the effectiveness of RAI versus reoperation when LNM were detected on ^131^I SPECT/CT after the initial RAI. RAI improving the outcome for patients with LNM is well known, as for example Guo et al. analysing 105,195 patients and Sun et al. analysing 15,953 patients showed before [19, 20] but both studies did not consider size and ^131^I avidity of LNM. Bouvet et al. analysed the impact of adjuvant RAI after reoperation in 85 patients with locoregional persistent or recurrent DTC and found no influence on recurrence-free survival [8], but did not included the impact of RAI without reoperation. In contrast, we analysed RAI vs reoperation after first RAI. Our results indicate that for small ^131^I positive LNM, the choice between reoperation and RAI does not affect the time to CR. However, PFS is longer following a second RAI compared to reoperation, a second RAI seems to be the more effective treatment. For patients with enlarged LNM, regardless of ^131^I avidity, the therapy does not affect PFS, but the likelihood of achieving CR and simultaneously becoming a low risk patient in the delayed risk stratification, increases significantly after reoperation. Hence, when enlarged LNM are detected on ^131^I SPECT/CT, reoperation appears to be the more effective treatment.

In this study, we focused solely on imaging based parameters to assess prognosis in patients with DTC, although biochemical markers such as Tg are commonly used in clinical practice. Especially pre-ablative Tg level is an established biochemical parameter and serves as an independent risk factor for persistent and recurrent diseases as well as for CR and overall survival [4, 21, 22]. However, for Tg levels in the range of 1–10 ng/ml, which often occur in patients with LNM and without distant metastases, no uniform further gradations exist for decision-making [4, 18]. Furthermore, differences in Tg levels occurring from exogen or endogen TSH stimulation are more significant. The rationale for the exclusion of Tg from our analysis was based on the specific aim of our study, which was to evaluate the prognostic value of imaging modalities in isolation, and our intention was to explore this approach without the potential confounding influence of biochemical markers. The exclusion of Tg data may limit the comprehensive understanding of patient outcomes, as it is well known that Tg levels can serve as an early marker for recurrence in some cases. Future studies could benefit from incorporating both imaging findings and biochemical markers such as Tg to provide a more holistic prognostic model. Nonetheless, this study highlights the potential of SPECT/CT alone as a prognostic tool.

### Limitations

Although we were able to include nearly 1000 patients in this monocentric study, the number of patients in the subgroup with enlarged ^131^I negative LNM was limited, with only 2 out of 22 patients undergoing reoperation. Therefore, no reliable conclusion can be drawn regarding therapy decisions for this subgroup. However, due to the monocentric design, our study cohort is homogeneous with respect to laboratory assays and surgical protocols. As with all retrospective studies, while initial treatments and subsequent diagnostic decisions during follow-up were similar, they were not based on a uniform prospective protocol. As far as it was possible in a retrospective analysis, the indication for the second therapy was noted. We could not consider further risk factors leading to the decision of reoperation or RAI. Other risk factors besides LNM might have been crucial for the decision and may influence the outcome too. Pathological ^131^I uptake was defined visually without further objective measurements. All post-therapy ^131^I SPECT/CT images were analysed by a single reader during clinical routine. An interobserver comparison was not performed, although all images were interpreted by an experienced nuclear medicine specialist. The definition of pathological ^131^I uptake compared to the salivary glands was used in order to establish a reliable reference organ, but this approach has not been used before in DTC. Since the CT scan was without contrast medium, contrast based pathological characteristics such as strong enhancement and heterogeneous enhancement could not be considered, which somewhat limits the diagnostic power of the CT imaging. Some studies, such as that of Adam et al., considered the number of pathological lymph nodes [23], whereas our study focused specifically on the size and ^131^I avidity of LNM.

## Conclusion

In clinical routine, it should be essential to conduct a precise evaluation of ^131^I SPECT/CT scans, as they provide critical information regarding patient outcomes and subsequent treatment plans. This is important since increased morbidity with reduced quality of life due to repeated surgeries can be avoided using this approach. Therefore, the post-therapy ^131^I SPECT/CT following initial RAI could be considered a first-time point for modified risk stratification. Despite the presence of small LNM, the prognosis remains favourable. Based on our results, ^131^I SPECT/CT can assist in the decision-making process regarding reoperation or further RAI.

## Supplementary Information

Below is the link to the electronic supplementary material.

Supplementary file1 (DOCX 17 KB)

## Data Availability

The datasets generated during and analysed during the current study are available from the corresponding author on reasonable request.
