# Supplementary material for: 131I SPECT/CT provides prognostic information in patients with differentiated thyroid cancer
Source: Eur J Nucl Med Mol Imaging. 2025 Mar 15;52(9):3170–9. doi: 10.1007/s00259-025-07187-1 (PMC12222370; doi:10.1007/s00259-025-07187-1)
Supplement: Supplementary file 1 — Supplementary file1 (DOCX 17 KB) [file 259_2025_7187_MOESM1_ESM.docx]

**Supplement Table 1** Therapy characteristics

|  | | | | CT0/S0 | CT0/S1 | CT1/S0 | CT1/S1 |
| --- | --- | --- | --- | --- | --- | --- | --- |
| Patients [n] | | | | 745 | 139 | 22 | 36 |
| Surgeries prior to first RAI | | | | | | | |
| One resection [n] (%) | | | | 481 (64,5) | 92 (66) | 16 (73) | 29 (81) |
| Two resections [n] (%) | | | | 262 (34,5) | 46 (33) | 6 (27) | 7 (19) |
| Three resections [n] (%) | | | | 2 (1) | 1 (1) | 0 | 0 |
| One-stage thyroidectomy [n] (%) | | | | 481 (65) | 93 (67) | 16 (73) | 29 (81) |
| Two-stage thyroidectomy [n] (%) | | | | 264 (35) | 46 (33) | 6 (27) | 7 (19 |
| LN resection | | Yes [n] (%) | | 352 (47) | 64 (46) | 11 (50) | 24 (67) |
|  |  |  | Central [n] | 24 | 3 | 5 | 13 |
|  |  |  | Lateral [n] | 3 | 7 | 1 | 6 |
|  |  |  | Central and lateral [n] | 6 | 18 | 16 | 5 |
|  |  |  | No declaration [n] | 9 | 1 | 0 | 0 |
|  |  | No [n] (%) | | 393 (53) | 76 (54) | 11 (50) | 12 (33) |
| Pre-ablative Tg level median [ng/ml] | | | | 2,90 | 4,70 | 14,80 | 21,40 |
| Second therapy | | | | | | | |
| Time between first and second therapy Median [months] (95% CI) | | | | 6,1 (7,36 – 10,95) | 6,0 (5,43 – 7,02) | 5,9 (4,25 – 8,99) | 4,2 (3,23 – 4,79) |
| Reoperation [n] | | | | 5 | 9 | 2 | 11 |
| Indication | | LNM [n] (%) | | 0 | 9 (100) | 2 (100) | 11 (100) |
|  |  | Residual thyroid tissue [n] (%) | | 5 (100) | 0 | 0 | 0 |
|  |  | Elevated Tg-level [n] (%) | | 0 | 0 | 0 | 0 |
| Reoperation | | | | 1 | 3 | 1 | 3 |
| Reoperation followed by RAI [n] (%) | | | | 4 | 6 | 1 | 8 |
| Second RAI [n] | | | | 226 | 49 | 13 | 14 |
| Indication | LNM [n] (%) | | | 7 (3) | 25 (51) | 2 (15) | 9 (64) |
|  | Thyroid remnants [n] (%) | | | 149 (66) | 11 (22) | 6 (46) | 3 (22) |
|  | Elevated Tg-level [n] (%) | | | 70 (31) | 13 (27) | 5 (38) | 2 (14) |

Abbreviations: LN: Lymph node; LNM: Lymph Node Metastases; RAI: Radioiodine therapy; Tg: Thyroglobulin
